# Smart Textiles and Sensorized Garments for Physiological Monitoring: A Review of Available Solutions and Techniques

**DOI:** 10.3390/s21030814

**Published:** 2021-01-26

**Authors:** Alessandra Angelucci, Matteo Cavicchioli, Ilaria A. Cintorrino, Giuseppe Lauricella, Chiara Rossi, Sara Strati, Andrea Aliverti

**Affiliations:** Dipartimento di Elettronica, Informazione e Bioingegneria, Politecnico di Milano, 20133 Milan, Italy; matteo.cavicchioli@mail.polimi.it (M.C.); ilariaanita.cintorrino@mail.polimi.it (I.A.C.); giuseppe1.lauricella@mail.polimi.it (G.L.); chiara9.rossi@mail.polimi.it (C.R.); sara.strati@mail.polimi.it (S.S.); andrea.aliverti@polimi.it (A.A.)

**Keywords:** sensorized garments, smart textiles, textile sensors, textile technologies, physiological monitoring, wearables

## Abstract

Several wearable devices for physiological and activity monitoring are found on the market, but most of them only allow spot measurements. However, the continuous detection of physiological parameters without any constriction in time or space would be useful in several fields such as healthcare, fitness, and work. This can be achieved with the application of textile technologies for sensorized garments, where the sensors are completely embedded in the fabric. The complete integration of sensors in the fabric leads to several manufacturing techniques that allow dealing with both the technological challenges entailed by the physiological parameters under investigation, and the basic requirements of a garment such as perspiration, washability, and comfort. This review is intended to provide a detailed description of the textile technologies in terms of materials and manufacturing processes employed in the production of sensorized fabrics. The focus is pointed at the technical challenges and the advanced solutions introduced with respect to conventional sensors for recording different physiological parameters, and some interesting textile implementations for the acquisition of biopotentials, respiratory parameters, temperature and sweat are proposed. In the last section, an overview of the main garments on the market is depicted, also exploring some relevant projects under development.

## 1. Introduction

The world of wearable devices is vast, and it is constantly expanding. It is intimately connected with people’s everyday lives, as anything functional to perform daily activities is actually “wearable”. Among all of them, wearables applied in the healthcare field are of major interest. Furthermore, the recent worldwide spread of COVID-19 is forcing several healthcare providers to rethink the way services are provided and is leading to a faster digitalization process [1], thus paving the way to a more intensive use of wearable devices and remote monitoring solutions in the medical field.

By combining the notion of biosensor and wearability, a useful and comfortable technology can be obtained. The market offers a great variety of wearables with different characteristics and monitoring approaches. These are connected in what can be considered a full-fledged network, commonly referred to as body sensor network (BSN) or body area network (BAN). A complete monitoring system implies the presence of an effective communication protocol and reliable data management and processing [2]. A smartwatch which measures a subject’s heart rate, sending data to an application in the user’s mobile phone that then processes all the information is a simple example of BSN [3,4,5].

The ultimate goal of these BSNs is the long-term monitoring of a subject without any constriction in time or space. The utilization of wearable sensors could satisfy multiple needs such as athletes’ performance evaluation, patient remote monitoring, and rehabilitation in a safe and accurate way [6], leading the concept of wearable device closer and closer to what is commonly referred as a medical device. Therefore, the main challenge is to succeed in combining the characteristics of these two families of devices. Wearable devices need to be comfortable, practical and should have an attractive design. Size and weight must match the intended use and simultaneously an adequate power supply and wireless communication must be introduced. Instead, medical devices must satisfy the safety and efficacy requirements, providing a precise measure of the physiological parameters. Moreover, the device needs to be certified (e.g., European Medical Device Regulation, Food and Drug Administration) so as to be produced and marketed in compliance with safety standards [3,4]. In other words, the product design must integrate well with the engineering; the simplicity, the usability and the relative cheapness of wearables should merge with the technological challenges yielded by medical purposes.

Nevertheless, several limitations must still be overcome, such as the technical limit regarding the duration of batteries, which must not represent a constraint in terms of size and shape; additionally, the power supply must grant enough usage time for a continuing monitoring [5,7]. Secondly, the large amount of circulating data introduced by the BSN itself leads to the problem of privacy. There are regional regulations regarding data ownership, such as the General Data Protection Regulation (GDPR) [8], but uniform worldwide regulations are lacking and this cannot be overlooked when thinking about a future where the use of wearable devices will be ordinary in medicine, sport, fitness, and more and more taking part of people’s lifestyle.

Moreover, all the components of the measurement system must be embedded in the same platform, making the ongoing monitoring as natural as wearing a pair of glasses or a garment. This is the basic idea behind the new generation of wearable systems, in which the monitoring devices are not only attached to the garment, but sensors and electronics are integrated into the garment itself. Therefore, the fusion of the textile world and biosensors represents the turning point of the new generation of wearables. Textile technologies, also called “electronic textiles”, or simply “e-textiles”, are fabrics made of filaments woven together in different ways that are capable of interacting with an external environment, such as the human body. They are also referred to as “smart textiles” to highlight the ability to accomplish functions that common clothes cannot fulfil [4] in various sectors, among which are healthcare [9] and industry [10]. Other commonly used terms include “intelligent fabrics”, “intelligent clothing”, “smart fabrics”, “wearable electronics”, and “textronics” [11,12].

In particular, the healthcare sector is the main driving force of the e-textiles market which is growing very fast. The 2015–2025 decade has been recognized as the “Wearable Era” and the market size is expected to exceed 5.55 billion USD in the next five years [5,13]. Sensorized garments are spreading very fast thanks to the technological advances in terms of miniaturization, which is essential for a non-intrusive monitoring.

E-textiles must meet different requirements, regardless of the manufacturing. The choice of the yarn’s material is fundamental because it affects both the production technique, that has to be adopted by the manufacturer, and the main properties of the sensing garment, i.e., sensors’ functioning, washability, elasticity, softness, adherence to the body and the final product’s life cycle. The integration of electronics into the fabric is the pivotal point of this technology. The intrinsic rigidity of conductive materials must match the normal wearing conditions, and this is being addressed with flexible electronics. Moreover, wearable sensors are subjected to mechanical deformations which can seriously compromise their sensing capability: the skin-sensor interface is one of the main problems that need to be addressed. 

The complete integration of sensors in the fabric leads to a well-defined industrial manufacturing process. It means that adding the sensors will mainly concern the weaving process, thus eliminating the further step of embedding them after the production of the garment. This represents a big advantage in terms of production costs [5]. Furthermore, the variety of different physiological parameters requires different measurement approaches to minimize the noise level, whose origin is multifactorial. Finally, if garments are used for energy harvesting and electricity generation as well as for physiological measurements, additional specifications are introduced in the manufacturing process [14].

While there are reviews focused only on the aspect of the materials composing the fabric [15,16] and others focused only on the physiological measurements that can be obtained, the purpose of the presented work is to provide the reader with a broader overview of all the aspects related to smart textiles for physiological monitoring, both in terms of manufacturing and application. For this reason, this review is intended to provide a detailed description of the textile technologies, describing the implementation of e-textile to make sensors for physiological parameters measurements. An additional focus is pointed at the technical challenges and the advanced solutions introduced with respect to conventional sensors. The presented paper is a focused literature review of current, high-quality articles in the field of smart textiles and sensorized garments, and information is obtained from scientific journals in various fields, including but not limited to biomedical engineering, electronics, materials science, information technology and mechanical engineering [17]. The articles have been searched on Google Scholar, Pubmed, Scopus, Google Patents, and Web of Science, using keywords like “garments”, “smart textiles”, “textile sensors”, “textile technologies”, or similar, and limiting the publication date to papers published after 2000.

Section 2 introduces the most common textile technologies from the point of view of manufacturing of conductive fibres, yarns, and inks. Throughout Section 3, applications of textile technologies employed to record biopotentials, respiratory parameters, and briefly temperature and sweat are discussed. Additionally, the section focuses on how the new e-textile systems deal with the technological, design, and measurement challenges, with respect to conventional technologies. In Section 4, an overview of the sensorized garments on the market is depicted, also exploring some relevant projects under development. Section 5 introduces to the latest frontiers in the field of research and is followed by a brief conclusion in Section 6.

## 2. Textile Technologies

**Figure 1:**
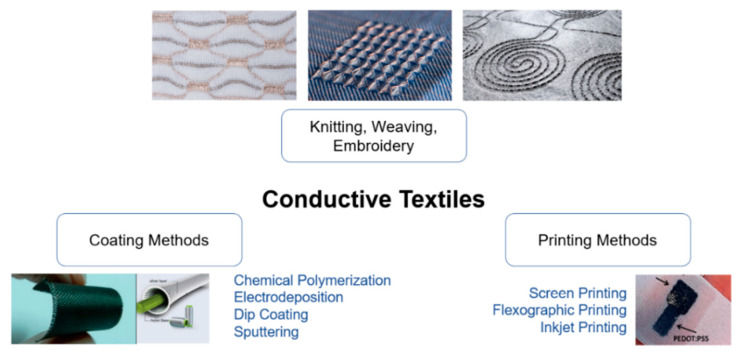
Most common manufacturing techniques of conductive textiles: knitting, weaving, embroidery; coating methods; printing methods.

From the microscopic to macroscopic scale, the fabric is composed of fibres and yarns. The former is the basic element of the textile material, whilst the latter is an intermediate material between fibre and fabrics, composed of interlocked fibres. Both the components can be made conductive applying different techniques [18], the most used of which are shown in Figure 1; their advantages and disadvantages are synthesized in Table 1 and further explained in the following sections.

### 2.1. Manufacturing of Conductive Fibres

There are two processes to make conductive fibres: wire drawing and fibre coating. Wire drawing is a mechanical process that transforms the raw material into microfilaments with a diameter of 5–8 mm, applying forces with industrial machines. After the drawing, the microfilament is annealed at a high temperature of 600–900 °C to restore its mechanical and electrical properties. Afterwards, the wire is cooled and wrapped in a revolving cylinder. The most used metals for this process are copper, silver, bronze, steel, and silver-plating copper, and their electrical properties are shown in Table 2. The obtained fibres yield several advantages. They are resistant to washing and sweat; the fibres are also strong, biologically inert, and available at low cost. Nevertheless, they are difficult to manufacture due to their heaviness [4].

Fibre coating consists of applying metals or conductive polymers on the surface of a non-metallic substrate to make it conductive [19]. The substrate can be either a fibre, a yarn, or a fabric. In the presented review, the fibre is considered as conductive unit for the purpose of clarity.

The different techniques used to make conductive fibres are sputtering, chemical polymerization, electrodeposition, and dip coating. The first two mentioned techniques, i.e., sputtering and chemical polymerization, are both classified as Physical Vapor Deposition (PVD) processes [23,24]. PVD is a coating process carried out in a vacuum in which thin films are deposited by the condensation of a vaporized form of the desired film material onto the substrate. The solid coating material is evaporated by heat or by bombardment with ions, such as in the case of sputtering; at the same time, the introduction of a reactive gas in the vacuum forms a compound with the metal vapor and is deposited on the substrate as thin film [25]. This describes what happens in sputtering, where the material is transformed from solid or liquid to vapor state and then it is carried by a vacuum or plasma system to the substrate on which it condenses. The conductivity of the final fibre depends on the type and thickness of the film coating material. The limitations of this technique concern the elevated production cost and non-trivial scalability of the vacuum processing.

Chemical polymerization is a PVD technique that allows the formation and deposition of intrinsically conductive polymers with a single step. The deposition is uniform, and the small thickness does not affect the mechanical properties of the fibres. The deposited films are wash and wear resistant. This technique shares the limitations of the previous one.

In electrodeposition, a fibre is immersed in a solution containing the ions of the metal with which the fibre will be coated. An electric current on a conductive material immersed in the same solution induces the deposition of the metal on the fibre [26].

Finally, in dip coating, the fibre is immersed in a solution containing conductive materials. After the removal of the excess material, a drying step (curing) permits the evaporation of the solvent and the fixation of the conductive particles on the fibre surface.

These coating techniques allow creating conductive substrate with good conductivity, maintaining the original fibre properties such as density, flexibility, and handiness. Moreover, they are resistant to corrosion, although the adhesion between the fibres and the metal might present some problems. Finally, the coated fibres are incorporated and twisted with non-coated fibres so as to form yarns [4].

In general, the fibres are composed of a core made of cotton, polyester and lycra, whilst the metals chosen for coating purposes are mainly silver, stainless steel and copper, whose characteristics are listed in Table 2 [4,21,22,27]. In particular, the information on the metals are reported with a higher level of detail in the works by Stoppa and Chiolerio [4] and by Kunal Singha et al. [20].

### 2.2. Manufacturing of Yarns

As far as the yarns are concerned, these can be manufactured in different ways to form the fabrics. The manufacturing processes are knitting, weaving, and embroidering; the resulting fabric is named after the used technique. The processes used for smart textiles are the same as those used for regular textiles.

A knitted fabric is made of a single yarn looped continuously to create a braid-shape and with the use of a needle, a series of yarns are connected. This manufacturing technology carries several advantages: skin comfort, low weight, and high elasticity. In fact, the knitted fabric is slightly more stretchable in width than in length, but if stretched too much it may lose its shape. Another problem concerns the intermittent contacts between the yarns that can create fluctuation in electrical resistivity [27].

Knitted fabrics provide stretchiness and temperature control, and thus, they are preferably employed for warmth, comfort, and wrinkle resistant applications, like clothing, although they shrink when frequently washed.

The knitting technology can be divided into weft and warp fabrics. Weft knitted fabrics, produced in flat or tubular form, are highly elastic and drapeable, while warp knitted fabrics are not much elastic while difficult to reveal [18,28]. 

On the other hand, the woven fabrics interlace two perpendicular sets of yarns that cross each other at right angles to form the grain. The fabric could loosen along its length, but never along its width [27]. Thanks to the high manufacturing tension, these fabrics are long-lasting and less likely to shrink when washing, as well as to lose their colour.

The knitted fabrics and woven fabrics are mass produced using the weft (or warp) knitting machine and the loom machine, respectively [18,27].

Another typology of fabric is the embroidery, a decoration of conductive patterns on finished textile surface. This technology is of interest in the field of smart textiles because of the possibility to lay the base material in all directions rather than in pre-defined ones [29]; because of this, an enhanced skin-electrode contact can be obtained [27].

Among all the fabrics, there are also non-woven textiles that show a random trend of the fibres, without identifying any ordered structure and privileged directions. Non-woven substrates are often made of high-quality fibres with versatile properties which can transform a two-dimensional substrate into a three-dimensional product. They function, often hidden away, as inserts, paddings, or as backings to give support to outer shell fabrics or three-layer assemblies or used as linings or insoles. Additionally, the surface structure, density, porosity, and thickness of non-woven fabrics can be controlled during the manufacturing process in a faster and cheaper way. For this reason, non-woven fabrics are more suitable for printing techniques, which will be discussed in the following sections. However, woven are more comfortable to wear as they are more flexible and breathable than nonwovens, which are temperature and humidity dependent [28].

All the manufacturing technologies described above (weaving, knitting and embroidery) have many disadvantages such as complex manufacturing processes, limitations in the type of fabrics, and inevitable damage of the natural properties of textiles. For this reason, printing technologies, such as screen printing, inkjet and flexographic, were developed to create conductive patterns on textile substrate allowing a reduction in production cost and a large-scale production. A drawback of printing methods is that the performance depends on ink penetration to the substrate, so it is optimized when the surface is smooth and flat, and ink is thus not dispersed into the internal surface of the substrate. Another challenge is the durability of the printed pattern with prolonged use [16].

The screen-printing method consists of using of fine meshes or stencils applied on a substrate. These have openings to enable the preferential passage of conductive inks only on the area of interest of the substrate. This method allows eco-friendly fabrication procedures and can be easily applied to various surfaces, such as plastics, paper, polymers, and textiles.

The inkjet printing method controls the accurate deposition of ink on different substrates, such as paper, glass, PET and polymer substrates. This printing method has a controllable nozzle to allow spraying the ink in the region of injection without a mask. Moreover, this technique allows a reduced production time.

Flexographic printing is a faster technique than the previous two and it works better with conductive inks. It is capable of high-level printing with a high resolution. Flexography is a direct rotary printing method, which uses raised matrix plates [27,30].

Various materials can be used to create conductive inks, however, the most largely used are the silver nanoparticles. Silver is the most suitable material for conductive circuits in printed electronic devices because of its high electrical conductivity, oxidation resistance, and good biocompatibility. It is also strong, unaffected by moisture, resonant, moldable, malleable and with highest thermal coefficient. Moreover, the silver printed fabrics are washable, although the repeating washing can reduce the conductivity substantially [30].

Conductive polymers, like polyaniline (PANI) and PEDOT:PSS, are used in all the mentioned techniques, as they are light, low cost, with good conductivity and mechanical flexibility. These organic polymers unite both metals’ electrical properties and plastics’ mechanical features [31].

They are suitable to produce electrically conductive yarns, antistatic coatings, electromagnetic shielding, and flexible electrodes. The most explored conductive polymer for conductive textiles applications is PEDOT:PSS. It can be directly polymerized or printed on the fabric or it can be used for fibres coating [22]. One of the first e-textiles, still used nowadays as a basis for the new prototypes in commercial industry, is the U.S. patent: “a composite elastic and wire fabric for physiological monitoring apparel” [32]. It was patented in 2002 and it is formed by elastic bands with conductive wire affixed following a curved pattern.

### 2.3. Applications of Stretchable and Conductive Inks

Conductive inks can be used for two main purposes: one is to realize electrodes and sensors themselves, while the other is to obtain the conduction of signals in the garment (conductive wires). 

A procedure to print conductive ink may be constituted by the following steps: printing an adhesive onto a compression fabric in a first pattern (the adhesive is elastic when dry); printing a conductive ink onto this first pattern; forming a gradient region between the conductive ink and the adhesive. This gradient region comprises a mixture of the conductive ink and the adhesive and the concentration of the ink in the gradient region decreases from a region closer to the layer of conductive ink to the layer of elastic adhesive [33]. There are specific techniques that make it possible to directly print a conductive ink onto a fabric, however this usually requires high temperatures. For this reason, there are research and industrial efforts to do so with nanoparticles at lower temperatures in order not to damage the fabric itself [34,35]. 

In terms of conductive wires, a limitation that is found in garments for the measurement of physiological parameters is that the leads providing power to and receiving signals from the sensors have not been fully integrated with the garment in a way that allows the garment to be flexible and comfortable [33]. A solution that has been found is to realize these connectors with the material SPIDON, a flexible fabric ribbon [36]. The L.I.F.E. shirt, which is described later in Section 4, is an example of sensorized garment that uses conductive ink both for ECG electrodes and connectors.

Furthermore, conductive inks printed atop textiles are vulnerable to cracking because of the deformable and porous structure of textiles. A solution that has been found to this problem consisted of controlling the ink permeation in the structure of the textile by adjusting the ink’s solvent [37].

## 3. Measurement of Physiological Parameters

**Figure 2:**
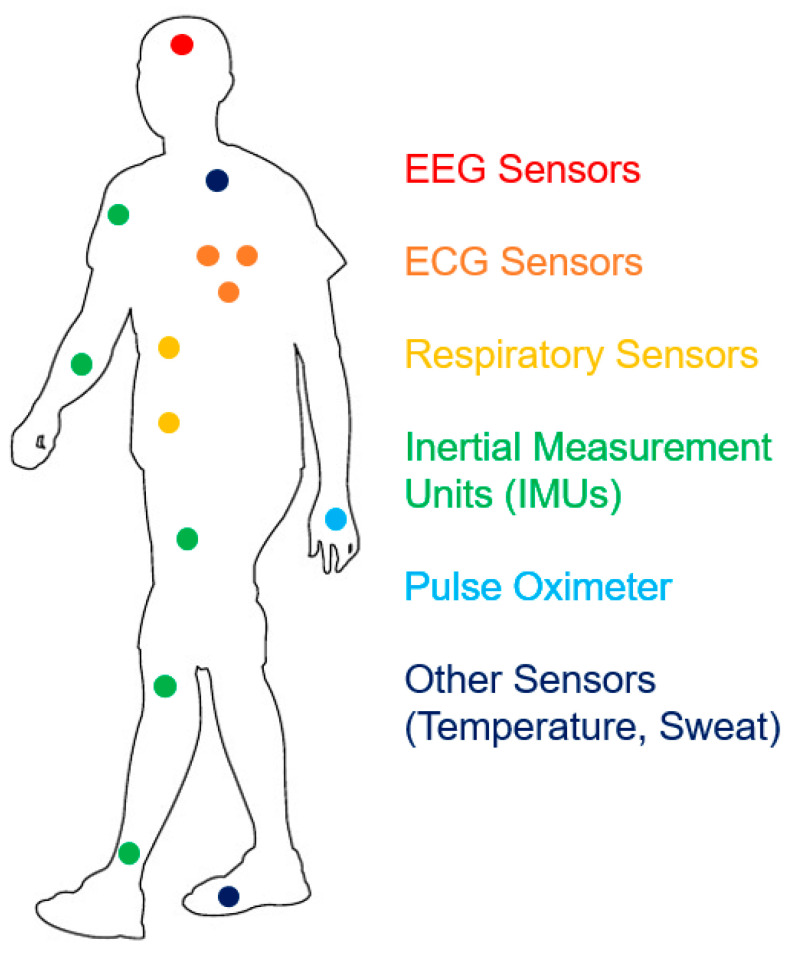
Typical placement sites of sensors that can be included in sensorized garments (ECGs, respiratory signal monitors, IMUs, pulse oximeters, other sensors).

Figure 2 shows some common placement sites of the monitors of different physiological parameters on a sensorized compression garment, which keeps the sensors in the needed positions to perform the needed measurements while avoiding movement artefacts. In fact, specific solutions have been developed to solve this problem: one example is a support mechanism that can be expanded in a direction perpendicular to the sensor/inner surface of the garment, where the support is backed by a supporting structure that is less flexible than the inner fabric of the garment, so that expanding the support puts the sensor in contact with the body [38].

In all the presented technologies, it must be noted that the benchmarking of the sensing performances is referred to single works presented in the literature, since different research projects obtained different results.

### 3.1. Biopotentials

Biopotentials are physiological electrical signals arising from the activity of electrically excitable cells, the detection of which, depending on the region, allows the monitoring of the cardiac activity (ECG), brain activity (EEG), and muscular activity (EMG) [5]. Their main characteristics are synthesized in Table 3 and later explained in Section 3.1. 

Traditionally, biopotentials are acquired using wet and disposable Ag/AgCl electrodes only in clinical or laboratory environments [39]. These electrodes are characterized by a gel layer to decrease the skin-electrode contact impedance, and an adhesive padding to increase the skin contact and reduce the motion artifacts. This system involves several disadvantages. The first disadvantage is the long setting time due to skin preparation and electrodes placement, which requires trained personnel.

**Figure 3:**
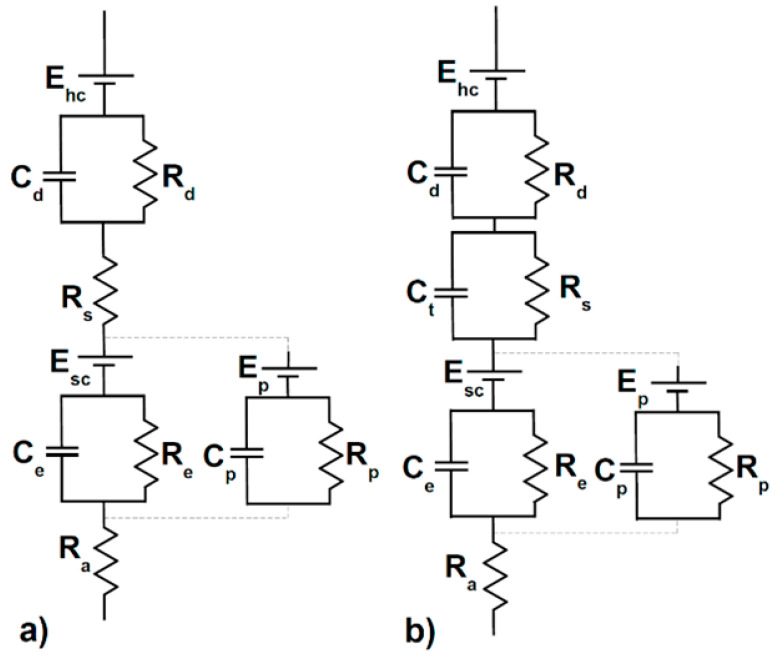
Equivalent circuit of the interface between skin and electrodes in the case of (**a**) wet electrodes and (**b**) textile electrodes. The figure was adapted from [27]. E_hc_ represents the electrode; C_d_ and R_d_ represent the impedance due to the sensor-electrolyte interface; C_t_ (only present in textile electrodes) and R_s_ represent an impedance due to the electrolyte layer; C_e_ and R_e_ represent the middle-layer impedance; C_p_ and R_p_ represent the sweat glands; R_a_ represents the inner-layer impedance, acting as a pure resistance in both cases.

The second disadvantage is that the gel materials might cause allergic reactions or irritations, and the third one is that wires constrain the movements resulting in an uncomfortable system [27]. Therefore, textile technologies are considered an appealing and valid alternative, introducing ‘dry’ electrodes to replace the traditional ‘wet’ electrodes. The most important advantages regard high comfort, no setting time, freedom of movement, and performing long measurements in any environment. Still, e-textile technologies entail some critical aspects such as high skin-electrodes contact impedance, sensitivity to motion artifacts [40]. Textile electrodes are devoid of the traditional gel layer, thus an additional impedance between the skin and the electrode is introduced, reducing the signal-to-noise ratio. In Figure 3, an equivalent circuit of the skin-electrode interface is proposed, depicting this effect.

The reported skin model is composed of an outer layer, modelled as a voltage generator E_sc_, as it acts like a semipermeable membrane causing a difference in ion concentration. An inner layer acts like a pure resistance, R_a_, and the middle layer impedance is modelled as a resistance R_e_ and capacitance C_e_ in parallel to another couple C_p_ and R_p_ which represent the sweat glands. Concerning the ‘wet’ electrode, the sensor-electrolyte interface is depicted as the parallel of resistance R_d_ and capacitance C_d_, and the electrolyte layer is only modelled as the resistance R_s_. In the ‘dry’ electrodes circuit, this last resistance is replaced by a significant impedance composed of the capacitance C_t_ and resistance R_s_, where C_t_ is inversely proportional to the skin moisture and sweat. Finally, the electrodes in both cases are represented by the potential E_hc_ [27]. The models of the skin and electrode-electrolyte interface [41] are based on the combination of equivalent circuits for the electrode-electrolyte interface [42] and equivalent circuits of the skin [43].

The overall impedance of wet electrodes can be computed as follows:

(1) $$Z_{\mathrm{wet}}=\frac{R_{d}}{1+{j\omega R}_{d}C_{d}}+R_{s}+\frac{R_{\mathrm{eq}}}{1+{j\omega R}_{\mathrm{eq}}C_{\mathrm{eq}}}+R_{a}$$

Considering R_eq_ and C_eq_ as the resistance and the capacitance obtained from the parallel of the middle-layer and the sweat glands in both models in Figure 3. In the case of textile electrodes, the previous formula becomes:

(2) $$Z_{\mathrm{textile}}=\frac{R_{d}}{1+{j\omega R}_{d}C_{d}}+\frac{R_{s}}{1+{j\omega R}_{s}C_{t}}+\frac{R_{\mathrm{eq}}}{1+{j\omega R}_{\mathrm{eq}}C_{\mathrm{eq}}}+R_{a}$$

Since, in comparison to standard metal electrodes, textile electrodes show a strong capacitive behaviour at the interface due to the lack of the hydrogel or electrode paste, which is often used as an electrolyte layer, this behaviour is compensated for by introducing a hydrogel membrane between the garment and the skin. The knitted pattern has shown to enhance the fibre-skin contact, a better tolerance to noise, and results to be more comfortable with respect to the woven pattern. Other factors affecting the skin-electrode impedance are the electrodes’ dimension: the bigger it is, the lower the contact impedance [27,44]; moreover, the sweat has proven to enhance the SNR. In any case, the skin-electrodes contact impedance is very difficult to evaluate due to the high inter-subject variability as well as intra-subject variability, i.e., hairy and non-hairy regions [45].

The second critical aspect concerns the susceptibility to motion artifacts, which depends on the size and tightness of the garment. The adhesion between electrodes and the skin is not guaranteed by the traditional adhesive padding, and depends on the fitting of the garment, thus, the artefact is difficult to address This noise is often confined by placing preamplifiers subsequently after the electrodes and using digital signal processing (DSP) algorithms [27].

#### 3.1.1. Electroencephalography

Electroencephalography (EEG) is a technique designed to monitor brain activity by recording the postsynaptic electrical signals non-invasively. The biopotentials are detected using surface electrodes placed on the scalp, and the recording is characterized by high temporal but low spatial resolution. EEG is used in several clinical applications, among which are diagnosis of neurological diseases and sleep monitoring, where e-textile solutions are introduced to provide accessible, comfortable, and long-term measurements [27]. A characteristic hindrance to EEG measurements consists of the high skin-electrode contact impedance due to the hair on the scalp, which is dealt with by measuring the biopotentials on the forehead with textile electrodes embedded in garments such as hairbands or hats, and some explanatory examples follow.

The first e-textile application concerns a set of electrodes manufactured with a seamless knitting technology in which a wire coated by Ag/AgCl is woven inside a nylon headband. The electrodes were placed on the forehead to avoid hair interference demonstrating good functionality on long measurements while maintaining constant and high comfort [46]. 

Another application consists of a set of electrodes fabricated by a printing technique. The electrodes are placed on a headband in which the circuit is printed through conductive ink and connected to the electrical components by silver loaded epoxy adhesive. The whole system is covered by a printed layer of dielectric and then by a printed layer of conductive material composed of carbon loaded rubber; an innovative element of this project concerns the power supply, based on solar panels [47]. To prove the reliability of this approach, the measurements were correlated with EEG recorded by standard Ag/AgCl electrodes during a real time emotion classification experiment. The comparison resulted in a correlation of 70.88% and an accuracy of 90 (±9)% [47].

In another work by Lin et al., a novel electrically conductive polymer fabric, which had 0.07 Ω/square of conductivity, was coated on a 0.2 mm thick taffeta material and benchmarked against standard electrodes, showing a high correlation of ~96% and ~90% from the forehead and hairy sites on the head [48].

Lastly, a case is described where the measurement system is composed of a set of polyaniline (PANI)-coated PU foam electrodes fabricated by an in situ aniline polymerization technique. Everything is covered by an insulating cotton fabric with a central hole to allow skin-electrode contact. The advanced characteristic of this research concerns the electrodes’ location, which is on the hairy scalp. Comparing the EEG in frontal position (FP2-F4) and on the scalp, the results were very similar, and this was possible thanks to the deformability of the foam electrode which was maintained in place by a Velcro with a certain compression. This mechanism allows the maintenance of very low contact impedance even without gel and for long-term measurements [49].

#### 3.1.2. Electromyography

The electromyography (EMG) is a technique used to assess the muscular activity by analysing the electrical signal driven from the central nervous system to activate muscles. The electromyogram allows the investigation of the complex cooperation of different muscular groups [39]. Textile technologies are introduced to acquire surface EMG for home-rehabilitation, telemonitoring, prosthesis control, athletes’ performances tracking in different environments and other applications [39,45,50,51,52].

The critical aspects regarding surface EMG measurements concern motion artifacts, skin-electrode impedance, which depends on the garment fit, and repeatability of the measurements due to changes in electrodes placement [44,45]. The motion artifact is counterbalanced by the big size of textile electrodes which target the activity of a whole muscle group, contrary to traditional electrodes that capture the activity of single muscular units [45]. Textile-based electrodes used in a study denoted comparable performance to Ag/AgCl electrodes when the diameter of the electrode was greater than 20 mm and the applied pressure greater than 10 mmHg, while the baseline noise showed a tendency to increase with decreasing electrode size [53].

The most conventionally used textile technology for EMG electrodes is based on conductive yarns. For example, Samner et al. in collaboration with SMARTEX [54] created a textile electrode based on a conductive yarn made of stainless steel fibres and elastane. This electrode is backed up by a more rigid material to enhance the skin contact and the adhesion of the electrode also during movement. The connection between electrodes and data storage unit is created by yarns composed of stainless-steel fibres coated with PVC [55]. In this case, the fabric and the conductive fibres were intertwined using a knitted pattern, whereas in other scientific words a different manufacturing technique, the embroidery, was used instead. This last allows a better customization in the design and shape of the electrodes compared to knitted patterns, therefore, embroidered electrodes better adapt to different application and monitoring scenarios [45].

Another application of stainless-steel conductive fibres to record EMG concerned the control of an active hand and wrist prosthesis. A high density EMG was recorded using a sleeve on which 100 electrodes are placed and grouped in four 5 × 5 grids [50]. This application is realized because of the employment of textile electrodes; in fact, in a clinical environment this system would require a long setting time and trained personnel for the set up and overview, whereas the wearable system permits discarding setting time and electrodes placement variability, and increased comfortability [50]. Moreover, the activity can be performed at home while being tele-monitored, increasing the frequency of rehabilitation and thus the successful rate.

The last example of EMG textile technology is based on an advanced technology. A conductive Ag-powder/fluoropolymer-based nanocomposite ink is jet-printed on both side of a porous substrate, creating a high performing textile sensor. The two-layers printing method introduces several advantages; the conductive ink coats the nanofibers in the inner fabric layers, enhancing the mechanical durability, conductivity and adhesion of conductors to the textile, in addition to separating the conductive traces from the skin contact as they grow on the two opposite sides of the fabric. This two-layer design has signed a new path for an innovative approach to build textile sensors to record biopotentials in general [40].

#### 3.1.3. Electrocardiography

The electrocardiography (ECG) is the most utilized technique to assess the cardiovascular system. Traditionally, surface electrodes are placed on the chest to detect abnormalities of the heart rhythm non-invasively or monitoring cardiac rehabilitation [56]. The parameters that are usually extracted are the P-QRS-T complex, the heart rate, and the heart rate variability, as the alteration of these quantities is associated with cardiovascular diseases such as atrial fibrillation, and atrioventricular block. Nonetheless, changing heart rhythms can only be detected in long-term recordings, yet traditional recording systems are highly uncomfortable and constrain normal movements. In this context, e-textile electrodes are appealing especially for the high comfort of long-term recordings.

In terms of benchmarking, the evaluation of ECG generally aligns with three levels: (1) Heart rate (HR), and heart rate variability (HRV); (2) QRS complexes comparison; (3) P, T waves and related segments for further examination. The same-time-different-location comparison with a calibrated device is a general method to evaluate the performance of a new device [57] and different examples can be found in the literature, showing good correlation results [58]. As an example, in the work by Yapici and Alkhidir [59], the comparison of the results of conventional electrodes with the textile ones showed that the signals conform very well in time domain and display an average cross-correlation of 88% for the entire waveform and a maximum of 97% was achieved between two P-QRS-T segments.

When analysing the textile technologies applied to make ECG electrodes, a wide variety of techniques is used [27]. Therefore, two e-textile applications that confront the issues regarding ECG recordings are proposed. The majority of e-textile systems is based on two and seldomly more leads to record ECG [56]. Yu et al. proposed a research where a 12-leads ECG measurement has been compared to a traditional Holter recording. The 12-leads are inferred from the combination of 10 textile electrodes based on electrically conductive fabric (Shieldex Med-tex P180, Statex, Bremen, Germany) [60]. Among the 12 leads, three are independent and nine are redundant, making the recording more reliable and less prone to motion artefacts, which are one of the most important sources of noise for e-textile ECG systems. Other strategies to reduce this noise are proposed, based both on hardware and software solutions [45].

The last application is used for both ECG and EMG recording and stimulation purposes. The electrodes are made of PEDOT:PSS which is brush-coated or printed on a fabric, and the region of deposition is fenced by a PDMS rubber-like layer that is previously printed [49,61]. This technique has good performances during long-term monitoring of dynamic activity, and the use of this polymer is spreading, as it demonstrates high stability in water and resistance to mechanical stress whilst maintaining conductive properties [31]. The gel layer decreases the skin-electrodes impedance and the motion artifacts are highly moderated.

### 3.2. Respiratory Parameters

Monitoring the respiratory system allows preventing and screening for several diseases such as lung pathologies, heart failure, cardiopulmonary decompensation, and sleep apnoeas, as the well-functioning of this apparatus depends on several factors. Different methods have been recently developed to track the respiratory parameters outside of the clinical environment, and sensorized clothes fit well in this context because of their comfort, practicality, and non-invasiveness [62]. To track the respiratory trend, it is possible to use the combination of two parameters related to breathing, oxygen saturation and minute ventilation [3]; information on the performed activity, available in sensorized garments equipped with inertial units, helps to understand whether a patient is experiencing activity-related fatigue or distress [63].

Peripheral blood oxygen saturation (SpO_2_) is a first indicator of a well-functioning lung as it is the portion of oxygen bond to haemoglobin in the blood; generally, it is recorded at the earlobe or fingers using pulse oximeter. Minute ventilation is defined in function of time, and it is determined by the parameters’ respiratory rate (RR) and tidal volume. 

Concerning the evaluation of oxygen blood saturation, Rothmaier et al. conducted a study on the feasibility of a textile pulse oximeter. They compared different manufacturing methods, woven and embroidered textiles, of PET fabrics with embedded PMMA optical fibres in a pair of gloves to determine which technique results in a better light transmission and receiving capability of the textile sensor. The woven fabric did not allow a proper transmission or reception of the light as the optical fibres were arranged parallel to the skin, and thus, requiring additional conditioning. Instead, the embroidered fabric resulted as more feasible, and with an SNR directly proportional to the number of optical fibres and the light intensity [64]. Another solution is proposed by Satharasinghe et al. who developed a system in which an LED and a photodiode were incorporated into the fabric in a practical and comfortable way [65]. The technology was tested for the measurement of heart rate (HR), but its usability was guaranteed for all measurements concerning the use of LEDs and photodiodes, including the measurement of oxygen saturation [65], as the opto-electronic performance of the two textile sensors resulted comparable to standard technologies. 

Following which, the evaluation of minute ventilation is considered, which is pursued by the quantification of the tidal volume. In the literature, e-textiles including respiratory sensors have been largely studied [51]. A limitation that is found in wearable devices based on the variation of the thoracic dimension is that measurements are acquired in one spot of the chest wall, i.e., only one degree of freedom is considered in the model [66], while it is known from the literature that the rib cage and the abdomen give different contributions to the tidal volume with changing postures [67]. For this reason, garments that can embed multiple respiratory sensors in different positions allow to obtain more accurate measurements. Pacelli et al. studied the integration of two piezo-resistors in the fabric, created with two different manufacturing methods: the first one by embroidering a conductive yarn, Belltron^®^9R1 (Aramid Hpm LLC., Hilton Head, SC, USA), and the second one by printing a conductive elastomer supplied by Wacker LTD. The two sensors were tested for the evaluation of respiratory activity, and then employed in the MyHeart project for the prevention of cardiovascular diseases. To evaluate the respiratory pattern, the change in chest volume, and therefore, the change in the resistance of the piezo-resistors due to the deformation of the tissue was detected, and the results were compared with a plethysmography measurement. In conclusion, both sensors guaranteed a wide area of linearity, and hence, a good evaluation of the volumetric change, assuming low motion artefacts between the sensors and the body [68]. Concerning the motion artefact, Messaddeq et al. proposed a solution, using an antenna sensitive to the volumetric variations of the chest, and thus, of the lungs. The spiral antenna was made of multi-material fibres consisting of polyimide-coated hollow-core silica glass capillaries in which a silver layer was deposited using the liquid state deposition technique; the sensor was subsequently integrated into a cotton t-shirt. The antenna had the lower return-loss at the resonance frequency of 2.45 GHz, the gain of 3.41 dB and the radiation pattern that was a combination of half-wave dipole and multiple-turns spiral antenna. The detection of the breathing rate relied on the change of the antenna geometry due to the fabric mechanical stretch, and to the change of dielectric properties of the torso during breathing. Both these mechanisms led to a shift of the antenna’s resonance frequency: 5% stretch entailing a 0.006 GHz frequency shift, and 3.6 mm lung offset leading a decreased dielectric constant yielding to 0.030 GHZ shift [69].

Another project is ProeTEX, which focuses on developing interoperability sensors to continuously monitor the physiological parameters of the emergency-disaster personnel. The measurement of SpO_2_ and minute ventilation was performed by a shirt which is directly in contact with the body. Minute ventilation recordings were pursued by a textile piezo-resistor and a wire-shaped piezoelectric transducer with high electromechanical sensitivity, and this last configuration increased the SNR making the signal detection more robust and reliable, although the average measurement error increases with increasing temperature. The performances of these sensors were compared with a gold standard measure performed by a commercial spirometer. In the same context, the SpO_2_ was recorded by an optical transducer, based on controlled-source electromagnetic (CSEM) technology and made of several couples of light transmitter and receivers. A built-in processor triggered the best-located transmitters to obtain the highest signal level [70].

There are also research projects that use fibre optic technologies to measure the respiratory rate. In particular, Fiber Bragg Grating (FBG) sensors are able to convert the physical movement of the thorax during inspiration and expiration into a wavelength shift. For this reason, this technique has been exploited in several research works [71]. 

Finally, several studies show a good agreement between the results obtained with the garments and previously validated methods. The work by Scilingo et al. [72] presents a comparison of performances of strain fabric sensors and a gold standard for the measurement of respiratory parameters, with positive results. Massaroni et al. [73] compared a smart garment with six piezoresistive elements with the results obtained with optoelectronic plethysmography [74]: the difference between the average respiratory frequency was always lower than 1% and 4% during quiet breathing and tachypnea, respectively. 

### 3.3. Temperature and Sweat

Among all the different physiological parameters, temperature is an important indicator of the physiological homeostasis. It normally ranges between 36.5 °C and 37.5 °C and it can change depending on several internal and external factors. The traditional sensors used for temperature sensing are resistance temperature detectors (RTDs), thermistors, and thermocouples [75]. However, these conventional and widely used technologies permit only periodic measurements, whereas several applications require continuous temperature monitoring, e.g., for new-borns’ care. This problem can be overcome with temperature sensing fabrics (TSFs), which involve different technologies. The most common are RTDs and Fiber Bragg Grating (FBG) sensors integrated into the fabric.

Among the traditional sensors, RTDs are proved to be the most suitable to integrate in a fabric. The design and the simple electronics allow an easy adaptation to the industrial scale. Copper, Nickel and Tungsten are typically wrapped with a mixture of cotton and polyester. The resistance-temperature relationship has a linear trend and the measurement can be performed thanks to a small current supply, which passing through the metal wires provides a voltage drop proportional to the temperature [75].

The FBG sensor is an optical fibre whose core contains an alternation of a periodic portion of material with a different refractive index. When the light propagates within the fibre, some wavelengths are lost, and others are transmitted. The returning light will present a wavelength shift indicative of the skin’s temperature [76]. The FBG sensor is made sensitive with a metal coating, whose periodicity will change accordingly to the temperature and consequently the light’s pathway inside the fibre will be modified. A further coating is applied to increase the sensitivity of the overall sensing element, composed by a plurality of FBG sensors. FBG sensors find applications also in pressure measurement together with capacitive sensors.

Finally, the detection of sweat is a possible application: a real time monitoring of its chemical composition could be useful in medicine and fitness, and it could help in the diagnosis of several pathologies such as cystic fibrosis. Moreover, a continuous measurement could be particularly useful for athletes to keep dehydration under control. The main problem when facing chemical sensors is the collection of the sample, and a textile application for sweat detection is non-trivial. The majority of the textile solutions are based on a handling system [77], which collects sweat thanks to a moisture wicking material. The sweat is collected from the skin surface and it is brought to the sensing area by a micro pump. Then, an optical detection system is used to perform the measurement, typically based on a colorimetric approach. However, it should be underlined that the described process still takes a time around 25–30 min before providing results [77]. This is the average time needed to produce enough sweat that then needs to saturate the acquisition layer.

## 4. Sensorized Garments

After revising some applications of textile technologies to monitor physiological parameters, here we bring attention to the sensorized garments on which the technologies are embedded. According to the area of application, garments can be divided in five groups [16], as garments for:

- Healthcare, to be applied for the monitoring of different health conditions.
- Sport, to be applied for the monitoring physiological parameters and the tracking of athletic performances during training or competitions.
- Fitness, to help the training of general consumers and to allow them to have a more comprehensive understanding of their wellbeing.
- Social, to facilitate users in leisure activities.
- Work, to support users during work activity, in terms of both performance and safety.

Although there are five categories, the only garments available on the market are mainly in the fields of health, sport and fitness. In Table 4, the main garments currently produced are listed, including type of product, company, fabric structure, sensed parameters, data communication protocol and placement of the e-module, if available. Even though the majority of garments are applied on the torso and upper limbs, there are also examples of sensing elements placed on the lower-limbs [78] and the head.

The system architecture includes both hardware and software items, and it is composed of several subunits: control, communication, location, power, storage, display, sensing, actuator and two supporting subsystems that are the interconnection and software.

The sensing and actuator units can be based on textile technologies connected to the electronic board where all the other electronic subunits are integrated, or non-textile, and then integrated in the electronic board too [18].

The on-body sensors data are transmitted to the nearest Personal Digital Assistant (PDA) through a short-range communication node based on a low power wireless system such as ANT+, NFC, or Bluetooth, and this last is the most used [102]. The PDA can be a smartphone, computer, or a Field Programmable Gate Array (FPGA) on which processing algorithms and the data storage system are held. Another communication node permits the transfer of data to a remote healthcare server [103].

Following this, three garments representing the health and sport/fitness categories are described, to exemplify the utilization of textile technologies in marketed items.

First, the Hexoskin t-shirt (Carré Technologies Inc., Montréal, Canada) provides a wearable health monitoring system measuring, for example, HR, maximal heart rate (HR_max_), heart rate at rest, heart rate variability (HRV), heart rate recovery (HRR), RR, tidal volume, oxygen consumption, movement, step count, cadence, stride, activity level, burnt calories, and sleep quality. The commercial t-shirt is composed of textile electrodes to obtain a single-lead ECG and an e-module including breathing and movement sensors, called Hexoskin Smart Device. This has 30 h+ of battery life rechargeable with a USB cable. The Vest is made of Knitted (73% micro polyamide, 27% elastane) fabric that is anti-bacterial, UV protective, quick dry, and washable. The Hexoskin device can be connected to the Hexoskin App with a Bluetooth protocol, allowing the user to set up the vest and visualize the collected data. The system is also compatible with the following mobile health apps: Apple Health App, Wear OS, MapMyRun, Runkeeper, Runtastic. The user can also visualize the data on Hexoskin Online Dashboard, whereas healthcare professionals, researchers and technicians can employ the advanced VivoSense analysis software to import/export data, batch processing, and to produce ready-to-publish graphs [81]. There are different models of the device and the costs can vary from 399$ to 579$ for a complete kit [104]. Additionally, a space-grade smart garment from the same company is available for medical research and space telemedicine applications (Astroskin); in this case, the sensors allows the acquisition of a 3-lead ECG, respiration, pulse oximetry, blood pressure, skin temperature and a three-axial accelerometer [82,83].

The second example concerns the company Sensoria that supports professional and amateur runners in training and coaching with addressed products. The available garments are smart socks [105], a bra, and a T-shirt [99] that are antimicrobial, machine washable, comfortable, and breathable. The socks have an integrated textile pressure sensor paired via Bluetooth with a detachable and rechargeable anklet. This monitors the user’s steps, walking time, distance, speed, calories, altitude, cadence, and foot landing technique, while exercising. The bra and the T-shirt provide an accurate and consistent heart rate monitoring. They work best with the E-modulo sensoria HRM (Heart Rate Monitor), that has a battery life of over 8 months and that tracks the performance progress of the user. It connects with Bluetooth Smart and ANT+ to the Sensoria Run 2.0 mobile app and the Sensoria Virtual Coach and has over 8 months of battery life. In addition, these products advise the professional runners about their running mechanics, the correct or incorrect running positions so they can improve their running style. The price range of the products of the Sensoria collection ranges from 119$ to 398$.

**Figure 4:**
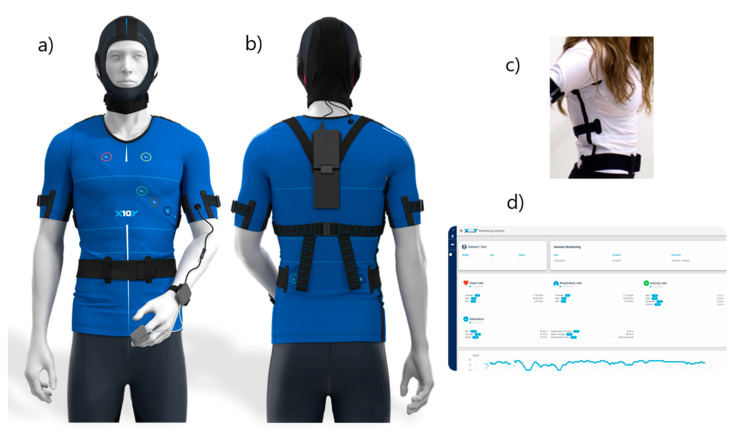
(**a**) Front and (**b**) back views of L.I.F.E.’s medical compression garment (BWell). The back view (right) shows where the plug is placed when the garment is worn. Both views show a cap to perform EEG which is currently being developed by the company. (**c**) An example of BWell’s fitting when worn. (**d**) Real-time data visualization dashboard. The figure was adapted from the website of the company producing the garment [106].

The third example is constituted by the smart shirt produced by Learn Inspire Free Entertain (L.I.F.E.) Italia Srl [106], which has been developed in two versions, one for sports and one for medicine. In particular, L.I.F.E.’s medical compression garment BWell (L.I.F.E. Italia Srl, Milan, Italy) is composed of 12 ink-based dry electrodes for ECG monitoring, five respiratory strain sensors and one accelerometer (Figure 4). The electrodes include a layer of adhesive, a layer of conductive ink, a binder, a solvent, a thickener and a gradient region between the conductive ink and the adhesive. The five respiratory sensors are positioned on the anterior surface of the garment and are made of an elastic ribbon impregnated with conductive ink, an electrical connector at each end of the elastic ribbon, and a cover made of compression fabric [84]. The version for sports Performer Wearware (L.I.F.E. Italia Srl, Milan, Italy), on the other hand, includes two ECG leads, two circumferential respiratory sensors and 10 accelerometers, since the focus is switched from health monitoring to performance monitoring. In this case, the garment includes not only a shirt but also shorts in order to monitor the movement of the subject’s thighs.

Among the products listed in Table 4, the sensorized garments Hexoskin/Astroskin (Carré Technologies Inc., Montréal, Canada), Zephyr^TM^ (Medtronic, Dublin, Ireland, Keesense (Chronolife SAS, Paris, France and BWell/Performer Wearware (L.I.F.E. Italia Srl, Milan, Italy) are the most indicated for respiratory monitoring, since they all include technologies that are specifically designed for the monitoring of the respiratory frequency. However, it must be noted that the Chronolife and Hexoskin garments only have a single-lead ECG, and Astroskin has a 3-lead ECG, while L.I.F.E.’s medical-grade solution has a 12-lead ECG, thus allowing for a comprehensive cardiac monitoring as well. Furthermore, shirts are the most performing in terms of cardiac monitoring because of the possibility to detect the ECG directly from the chest. Finally, there are some garments that only measure one parameter, such as Siren’s socks to detect foot temperature, and are therefore useful for the monitoring of specific diseases (e.g., in the case of Siren’s product, diabetes and its effects of limbs).

## 5. New Frontiers of Smart Garments

Up until now, there have been no commercially available devices that monitor the vital parameters of workers. Devices belonging to this category are yet under development, and an interesting example is provided. The PROeTEX project developed emergency disaster personnel smart garments based on an advanced E-Textile system. All the three developed prototypes, reserved to civil protections, urban and forest firefighters, are based on a shirt or inner garment (IG), a jacket or outer garment (OG) and a pair of boots. The IG continuously monitors physiological measurements like HR, breathing movement, cardiac sounds, and health-state parameters such as sweat, dehydration, electrolytes, stress indicators, O_2_, CO, and internal temperature thanks to textile sensors and electrodes that are directly in contact with the skin. The OG and boots, instead, measure activity and external chemical environment, including toxic gases and vapours and external temperature. The OG hosts also the PEB, professional electronic box, containing multiple sensors like accelerometers, piezoelectric sensors, and GPS module sensor, and collects all the data obtained from the three garments. Moreover, thanks to a transmission system composed of two textile antennas and an embedded PC board, the PEB transfers all the information to the local coordination workstation that is usually near the operative area via Wi-Fi.

The associated monitoring software allows visualizing the parameters related to each operator as well as his/her position: in the event of imminent danger, such as the presence of toxic gases, an immediate alarm is launched to the intervention managers that coordinates all the rescuers. Another long-range communication occurs from the local post to the central emergency coordination [70,107].

An additional field of research that is becoming important is the cardio-respiratory monitoring of children. Recently, new research has been accruing interest in the development of special garments for infants and children. However, it should be stressed that this market area has only grown over the past 10 years [18].

As it was previously mentioned, the Astroskin smart shirt is an example of wearable monitoring system that is usable in space telemedicine applications too [83].

**Figure 5:**
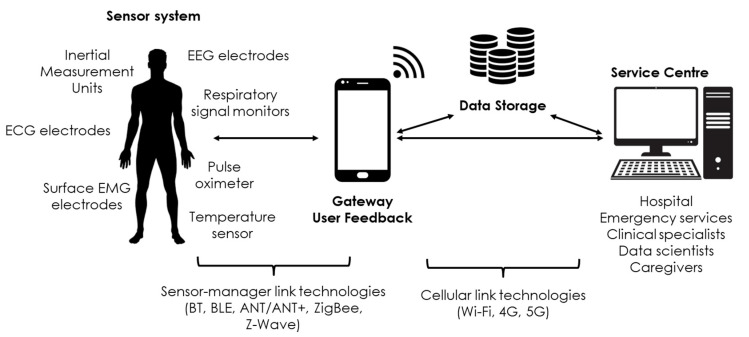
Telemonitoring system with a two-hop data transmission architecture. The figure was adapted from [2].

Furthermore, the advent of new mobile communication technologies, such as 5G, opens the door to a more systematic use of wearable sensors in general, and sensorized garments in particular, both for telemedicine and sports applications [108]. More specifically, the 5G communication enhances the acquisition of data from several sensors in parallel and the opportunity to scale the previously introduced solution to great cohorts of patients or healthy subjects without losses in the performance. Given the increased bandwidth that can be obtained with 5G, the fact that a garment usually includes several sensors does not constitute a problem. A typical architecture of a 5G-enabled telemonitoring system is the so-called two-hop architecture [102] and is shown in Figure 5.

Finally, flexible epidermal electronic technologies constitute a new frontier of electrodes for garments: they are both transparent and resistant to different types of mechanical deformation more effectively than fabric electrodes [109]. Thanks to their characteristics, continuous and long-term tracking of vital physiological signals, such as heart rate, artery pulse pressure and temperature, blood flow, and blood oxygen, can be realized during daily activities in a manner that is mechanically invisible to users.

## 6. Conclusions

Textile technologies are utilized for sensorized garments to monitor several physiological parameters in an advanced and innovative way, by performing long recordings in any environment, and with high comfort. Textile technologies are based on the concept of conductive fibres whose manufacturing techniques affect the recording and the performance of the fabric itself. Among the manufacturing processes, there are more traditional techniques such as coating, but also advanced techniques such as printing. In this field a lot of research is focusing on developing new biocompatible, robust, and sensitive materials. When it comes to measuring physiological signals, the textile sensors must meet technological challenges and requirements. Hence, the manufacturing process, the technology, and materials must be carefully chosen according to the application and the field of use. In most of the cases the reliability and feasibility of the measurements is yet to be confirmed with respect to the conventional techniques, and this field of research is vast and still developing. Finally, the observation is taken to the world of sensorized garments. Three garments are described, as they pool together most of the features characterizing these wearable devices thanks to the variety of performing measurements and field of use. We observe that the frontiers in this fields concern the development of garments for children as few companies fabricate garments for this category. To conclude, the innovations carried by the employment of textile technologies for sensorized garments are many, featuring a new generation of wearable devices. Nevertheless, a huge effort is yet focused on the research and development stage of this field.

## Figures and Tables

**Table 1:** Synthesis of the main advantages and disadvantages of the manufacturing techniques.

	Advantages	Disadvantages
Knitting	skin comfort; low weight; high elasticity	Complex manufacturing process; limitations in the choice of fabrics; damaging of the natural properties of textiles
Weaving	long-lasting fabrics; less likely to shrink when washing; less likely to lose colour	
Embroidery	possibility to lay the base material in all directions rather than in pre-defined ones (enhanced skin-electrode contact)	
Coating methods	good conductivity; maintenance of the original fibre properties such as density, flexibility, and handiness; resistant to corrosion.	high production cost; difficult to scale production
Printing methods	reduction in production cost; possibility of a large-scale production	durability of printed patterns; optimal performance achieved only with smooth and flat surfaces

**Table 2:** Materials used to manufacture e-textiles [4,20,21,22].

Metal (Monofilament Fibers)	Electrical Properties					Notes
	Conductivity [S·m/mm^2^]	Resistivity [Ω·mm^2^/m]	Thermal Coefficient of Resistance [10^−6^ K^−1^]			
			Minimum	Typical	Maximum	
Cu	58.5	0.0171	3900	3930	4000	Corrosion with water
Cu/Ag	58.5	0.0171	3900	4100	4300	-
Ag 99%	62.5	0.0160	3800	3950	4100	Biocompatible, stable, rare, unaffected by moisture, resonant, moldable, malleable
AgCu	57.5	0.0174	3800	3950	4100	-
Bronze	7.5	0.1333	600	650	700	-
Steel 304	1.4	0.7300	-	1020	-	-
Steel 316	1.3	0.7500	-	1020	-	-
Conductive polymer	Conductivity [S/cm]	Doping	Limitations			Advantages
PEDOT:PSS	4700	P	Brittle, needs additional steps to process			High conductivity, resistance to humidity, stable at high temperatures, transparent
PANI (Polyaniline)	112	P	Hard to process, not biodegradable, limited solubility			High conductivity, stable in different environments, low cost
Fabrics	Category	Relative permittivity (ε_r_) at 1 kHz				Physical characteristics
Cotton	Natural	3.004				Absorbent, breathable
Nylon (Polyamide)	Synthetic	1.222				Breathable, elasticity
Polyester threads (PES)	Synthetic	1.178				Breathable
Linen	Natural	4.007				Absorbent, breathable
Rayon	Synthetic	5.082				Breathable, elasticity, transparent
PVC textile	Synthetic	3.118				Waterproof, non breathable

**Table 3:** Synthesis of the main characteristics of biopotentials and used electrodes.

	Electroencephalography (EEG)	Electrocardiography (ECG)	Electromyography (Surface EMG)
Main applications	Sleep monitoring; diagnosis of neurological diseases	Assessment of heart rate variability; cardiovascular disease monitoring; cardiac rehabilitation monitoring	Movement tracking; telerehabilitation; prostheses control
Textile technologies reported in the literature	Knitting, printing	Knitting, printing, coating	Knitting, printing
Issues	Skin-electrode contact impedance affected by the presence of a hairy scalp	Number of achievable leads; motion artifacts	Motion artifacts; variability in skin-electrode contact impedance

**Table 4:** Overview of some garments available on the market or in research settings [79].

Base Products	Company and Name of the Product	Knitted Fabric Material	Sensed Parameters	Data Communication Strategy	e-Module Placement	Reference
T-shirt, vest	Biodevices, Biomedical Engineering Systems, S.A.	80% polyamide, 20% elastane	HR, HRV, ECG, movement	Bluetooth (VJ holter pro, Vital Jaket^®^)	Plug inside shirt pocket	[80]
Shirt (T-shirt, sleeveless shirt)	Carré Technologies Inc. (Hexoskin)—Hexoskin shirt	73% micro polyamide, 27% elastane	HR, HRV, HR recovery (HR2), RR, step count, cadence, stride, activity level, calories burned, sleep assessment)	Bluetooth (Hexoskin, Hexoskin X, Apple Health App, Wear OS, MapMyRun, Runkeeper, Runtastic)	Plug inside vest pocket	[81]
Shirt	Carré Technologies Inc. (Hexoskin)—Astroskin shirt	-	3-lead ECG, respiration (RR, minute ventilation, tidal volume), pulse oximetry, systolic blood pressure, skin temperature, 3-axial accelerometry	Bluetooth (dedicated smartphone and web app)	Plug inside vest pocket	[82,83]
Shirt, pants	L.I.F.E. Italia s.r.l.	-	12-lead ECG, 5 sensors to detect RR, accelerometry (activity level)	Bluetooth (L.I.F.E. app)	Plug inside the shirt, on the back	[84]
Shirt	Chronolife SAS-KeeSense	-	Thoracic and abdominal respiration, physical activity, single-lead ECG, temperature, pulmonary impedance	Bluetooth (Chronolife app)	Plug attached to the shirt, not to be removed when washing	[85]
T-shirt, vest, bra, sport bra	Emglare Inc.	100% recycled polyester	HR, ECG	Bluetooth (Emglare Heart, Apple Heart, Google Fit)	Integrated into the inside of the clothing	[86]
Vest	HealthWatch Ltd.	-	HR, ECG, skin temperature, RR, body posture	Wi-Fi, 3G, 4G (Master Caution^®^)	On the outer surface above the left side of the waist	[87]
Socks	Siren	-	Foot temperature	Bluetooth (Siren app)	Above the ankle	[88]
Vest, T-shirt, sport bra	AiQ Smart Clothing	-	HR, ECG, RR, temperature	Bluetooth, Wi-Fi	Over left chest	[89]
Underwear, customized smart textiles	Skiin Connected Innerwear, Myant Inc.	-	HR, temperature, pressure, motion, body fat and hydration levels	Bluetooth (SKIIN)	Slides into waistband	[90]
Shirt, cap	Bioserenity-Neuronaute	-	EEG, ECG	Bluetooth (Neuronaute app)	Attached on the top of the head (cap)/on the shoulder (shirt)	[91]
T-shirt	Bioserenity- Cardioskin™	-	12-lead ECG	Wi-Fi	Attached at the level of the abdomen	[92]
Vest	Smartex s.r.l.	-	Single-lead ECG, respiratory signal, 3-axial accelerometer	Bluetooth and Wi-Fi	Ventral top right part of the chest	[93]
Shirt, shorts, leggings	Athos	-	sEMG, HR, calories expenditure, active time/rest time	Bluetooth (Athos^®^ iOS app)	At the level of the sternum in the shirt, on the thigh in shorts and leggings	[94]
T-shirt, shirt, sports bra, strap	Medtronic-Zephyr™ Performance Systems	88% PES, 12% Spandex	ECG, RR, temperature, accelerometric data, time and location	BLE (OmniSense™ app)	Plugs into the garments	[95]
Vest	Polar Electro	-	Motion sensor, HR, GPS	Bluetooth (Polar Flow app)	Slides into the garment at the centre back of the neck	[96]
Compression sleeve	Komodo Technologies, Inc.	-	ECG/EKG, HR, HRV, SpO_2_, activity intensity (MET)	Bluetooth (AIO Sleeve)	Wrist	[97]
Shorts	Myontec Ltd.	71% polyamide, 29% elastane	EMG	Bluetooth (MBody Live 3 app)	At the level of the waist	[98]
T-shirt, socks, bra	Sensoria	polyamide 70–90%, elastane 5–8%, polyester 0–18%	HR, step count, cadence, stride, calories, altitude, distance	Bluetooth (Sensoria Fitness App, third party apps)	Connect to snap buttons under chest, fold sock over anklet	[99]
Yoga pants	Wearable X	-	Accelerometers, haptic feedback	Bluetooth (Nadi X app)	Clip into the pants behind the left knee	[100]
Sport bra	SUPA (based on Movesense platform)	95% PES, 5% lycra	HR, motion, temperature	Bluetooth (SUPA.AI app)	Under bust band	[101]

## Data Availability

Data sharing not applicable.

## References

[1] Adans-Dester C.P., Bamberg S., Bertacchi F.P., Caulfield B., Chappie K., Demarchi D., Erb M.K., Estrada J., Fabara E.E., Freni M. (2020). Can mHealth technology help mitigate the effects of the COVID-19 pandemic?. IEEE Open J. Eng. Med. Biol..

[2] Angelucci A., Aliverti A. (2020). Telemonitoring systems for respiratory patients: Technological aspects. Pulmonology.

[3] Aliverti A. (2017). Wearable technology: Role in respiratory health and disease. Breathe.

[4] Stoppa M., Chiolerio A. (2014). Wearable electronics and smart textiles: A critical review. Sensors.

[5] Andreoni G., Standoli C.E., Perego P. (2016). Defining requirements and related methods for designing sensorized garments. Sensors.

[6] Kańtoch E. (2018). Recognition of sedentary behavior by machine learning analysis of wearable sensors during activities of daily living for telemedical assessment of cardiovascular risk. Sensors.

[7] Cappellieri A., Henchoz N., Tenuta L., Testa S. (2020). Fashion wearable between science and design, from the product to an overall user experience. Int. J. Lit. Arts.

[8] Polkowski Z. (2019). The method of implementing the general data protection regulation in business and administration. Proc. 10th Int. Conf. Electron. Comput. Artif. Intell. ECAI 2018.

[9] Coyle S., Diamond D. (2016). Medical applications of smart textiles. Advances in Smart Medical Textiles.

[10] Cherenack K., Van Pieterson L. (2012). Smart textiles: Challenges and opportunities. J. Appl. Phys..

[11] Rambausek L. (2014). Textronics: Definition, Development and Characterization of Fibrous Organic Field Effect Transistors 2014.

[12] Gandolfo P. Bluetooth Low Energy, Zigbee, and Cognitive 3D-ICs Add Muscle to Telehealth. https://www.eetimes.com/bluetooth-low-energy-zigbee-and-cognitive-3d-ics-add-muscle-to-telehealth/#.

[13] Stylios G.K. (2020). Novel smart textiles. Materials.

[14] Chen G., Li Y., Bick M., Chen J. (2020). Smart textiles for electricity generation. Chem. Rev..

[15] Islam G.M.N., Ali A., Collie S. (2020). Textile sensors for wearable applications: A comprehensive review. Cellulose.

[16] Ghahremani Honarvar M., Latifi M. (2017). Overview of wearable electronics and smart textiles. J. Text. Inst..

[17] Mokhtari F., Cheng Z., Raad R., Xi J., Foroughi J. (2020). Piezofibers to smart textiles: A review on recent advances and future outlook for wearable technology. J. Mater. Chem. A.

[18] Sayem A.S.M., Teay S.H., Shahariar H., Fink P.L., Albarbar A. (2020). Review on smart electro-clothing systems (SeCSs). Sensors.

[19] Alagirusamy R., Eichhoff J., Gries T., Jockenhoevel S. (2013). Coating of conductive yarns for electro-textile applications. J. Text. Inst..

[20] Singha K., Kumar J., Pandit P. (2019). Recent advancements in wearable & smart textiles: An overview. Mater. Today Proc..

[21] Ng C.L., Reaz M.B.I. (2017). Characterization of textile-insulated capacitive biosensors. Sensors.

[22] Tseghai G.B., Mengistie D.A., Malengier B., Fante K.A., Van Langenhove L. (2020). PEDOT:PSS-based conductive textiles and their applications. Sensors.

[23] Korzeniewska E., Tomczyk M., Walczak M. (2020). The influence of laser modification on a composite substrate and the resistance of thin layers created using the PVD process. Sensors.

[24] Dietzel Y., Przyborowski W., Nocke G., Offermann P., Hollstein F., Meinhardt J. (2000). Investigation of PVD arc coatings on polyamide fabrics. Surf. Coatings Technol..

[25] Shahidi S., Moazzenchi B., Ghoranneviss M. (2015). A review-application of physical vapor deposition (PVD) and related methods in the textile industry. Eur. Phys. J. Appl. Phys..

[26] Basile F., Benito P., Fornasari G., Monti M., Scavetta E., Tonelli D., Vaccari A., Gaigneaux E.M., Devillers M., Hermans S., Jacobs P.A., Martens J.A., Ruiz P.B.T. (2010). A novel electrochemical route for the catalytic coating of metallic supports. Scientific Bases for the Preparation of Heterogeneous Catalysts.

[27] Acar G., Ozturk O., Golparvar A.J., Elboshra T.A., Böhringer K., Kaya Yapici M. (2019). Wearable and flexible textile electrodes for biopotential signal monitoring: A review. Electronics.

[28] Van Langenhoven L. (2007). Smart Textiles for Medicine and Healthcare: Materials, Systems and Applications.

[29] Mecnika V., Hoerr M., Krievins I., Jockenhoevel S., Gries T. (2015). Technical embroidery for smart textiles: Review. Mater. Sci. Text. Cloth. Technol..

[30] Hong H., Hu J., Yan X. (2019). UV Curable conductive ink for the fabrication of textile-based conductive circuits and wearable uhf rfid tags. ACS Appl. Mater. Interfaces.

[31] Grancarić A.M., Jerković I., Koncar V., Cochrane C., Kelly F.M., Soulat D., Legrand X. (2018). Conductive polymers for smart textile applications. J. Ind. Text..

[32] Istook C.L. (2001). Composite Elastic and Wire Fabric for Physiological Monitoring Apparel. U.S. Patent.

[33] Longinotti-buitoni G., Aliverti A. (2014). Methods of Making Garments Having Stretchable and Conductive Ink. U.S. Patent.

[34] Jo Y.H., Jung I., Choi C.S., Kim I., Lee H.M. (2011). Synthesis and characterization of low temperature Sn nanoparticles for the fabrication of highly conductive ink. Nanotechnology.

[35] Wang Z., Wang W., Jiang Z., Yu D. (2016). Low temperature sintering nano-silver conductive ink printed on cotton fabric as printed electronics. Prog. Org. Coat..

[36] Longinotti-buitoni G., Aliverti A., Pallai F. (2016). Flexible Fabric Ribbon Connectors for Garments with Sensors and Electronics. U.S. Patent.

[37] Jin H., Matsuhisa N., Lee S., Abbas M., Yokota T., Someya T. (2017). Enhancing the performance of stretchable conductors for e-textiles by controlled ink permeation. Adv. Mater..

[38] Longinotti-buitoni G., Aliverti A. (2016). Physiological Monitoring Garments with Enhanced Sensor Stabilization. U.S. Patent.

[39] Papaiordanidou M., Takamatsu S., Rezaei-Mazinani S., Lonjaret T., Martin A., Ismailova E. (2016). Cutaneous recording and stimulation of muscles using organic electronic textiles. Adv. Healthc. Mater..

[40] La T.G., Qiu S., Scott D.K., Bakhtiari R., Kuziek J.W.P., Mathewson K.E., Rieger J., Chung H.J. (2018). Two-layered and stretchable e-textile patches for wearable healthcare electronics. Adv. Healthc. Mater..

[41] Beckmann L., Neuhaus C., Medrano G., Jungbecker N., Walter M., Gries T., Leonhardt S. (2010). Characterization of textile electrodes and conductors using standardized measurement setups. Physiol. Meas..

[42] McAdams E.T., Jossinet J. (2000). Nonlinear transient response of electrode—electrolyte interfaces. Med. Biol. Eng. Comput..

[43] Webster J.G. (2009). Medical Instrumentation: Application and Design.

[44] Finni T., Hu M., Kettunen P., Vilavuo T., Cheng S. (2007). Measurement of EMG activity with textile electrodes embedded into clothing. Physiol. Meas..

[45] Shafti A., Ribas Manero R.B., Borg A.M., Althoefer K., Howard M.J. (2017). Embroidered electromyography: A systematic design guide. IEEE Trans. Neural Syst. Rehabil. Eng..

[46] Fleury A., Alizadeh M., Stefan G., Chau T. (2018). Toward fabric-based EEG access technologies: Seamless knit electrodes for a portable brain-computer interface. Proceedings of the 2017 IEEE Life Sciences Conference (LSC).

[47] Matiko J.W., Wei Y., Torah R., Grabham N., Paul G., Beeby S., Tudor J. (2015). Wearable EEG headband using printed electrodes and powered by energy harvesting for emotion monitoring in ambient assisted living. Smart Mater. Struct..

[48] Lin C., Liao L., Liu Y., Wang I., Lin B., Chang J. (2011). Novel dry polymer foam electrodes for long-term EEG measurement. IEEE Trans. Biomed. Eng..

[49] Muthukumar N., Thilagavathi G., Kannaian T. (2015). Polyaniline-coated foam electrodes for electroencephalography (EEG) measurement. J. Text. Inst..

[50] Farina D., Lorrain T., Negro F., Jiang N. High-density EMG E-textile systems for the control of active prostheses. Proceedings of the 2010 Annual International Conference of the IEEE Engineering in Medicine and Biology.

[51] Choudhry N.A., Rasheed A., Ahmad S., Arnold L., Wang L. (2020). Design, Development and characterization of textile stitch-based piezoresistive sensors for wearable monitoring. IEEE Sens. J..

[52] Di Giminiani R., Cardinale M., Ferrari M., Quaresima V. (2020). Validation of fabric-based thigh-wearable EMG sensors and oximetry for monitoring quadricep activity during strength and endurance exercises. Sensors.

[53] Kim S., Lee S., Jeong W. (2020). Emg measurement with textile-based electrodes in different electrode sizes and clothing pressures for smart clothing design optimization. Polymers.

[54] Chandler D.L. (2017). Making clothing smarter: Rita Paradiso of Smartex is engineering clothes that can monitor a wearer’s condition. IEEE Pulse.

[55] Sumner B., Mancuso C., Paradiso R. Performances evaluation of textile electrodes for EMG remote measurements. Proceedings of the 2013 35th Annual International Conference of the IEEE Engineering in Medicine and Biology Society (EMBC).

[56] Vojtech L., Bortel R., Neruda M., Kozak M. (2013). Wearable textile electrodes for ECG measurement. Adv. Electr. Electron. Eng..

[57] Sun Y., Yu X.B. (2016). Capacitive biopotential measurement for electrophysiological signal acquisition: A review. IEEE Sens. J..

[58] Seoane F., Soroudi A., Lu K., Nilsson D., Nilsson M., Abtahi F., Skrifvars M. (2019). Textile-friendly interconnection between wearable measurement instrumentation and sensorized garments—Initial performance evaluation for electrocardiogram recordings. Sensors.

[59] Yapici M.K., Alkhidir T.E. (2017). Intelligent medical garments with graphene-functionalized smart-cloth ECG sensors. Sensors.

[60] Yu X., Boehm A., Neu W., Venema B., Marx N., Leonhardt S., Teichmann D. (2017). A wearable 12-lead ECG T-shirt with textile electrodes for unobtrusive long-term monitoring–Evaluation of an ongoing clinical trial. EMBEC & NBC 2017.

[61] Takamatsu S., Lonjaret T., Crisp D., Badier J.M., Malliaras G.G., Ismailova E. (2015). Direct patterning of organic conductors on knitted textiles for long-term electrocardiography. Sci. Rep..

[62] Massaroni C., Nicolò A., Lo Presti D., Sacchetti M., Silvestri S., Schena E. (2019). Contact-based methods for measuring respiratory rate. Sensors.

[63] Angelucci A., Kuller D., Aliverti A. (2020). Respiratory rate and tidal volume change with posture and activity during daily life. Eur. Respir. J..

[64] Rothmaier M., Selm B., Spichtig S., Haensse D., Wolf M. (2008). Photonic textiles for pulse oximetry. Opt. Express.

[65] Satharasinghe A., Hughes-Riley T., Dias T. Photodiode and LED embedded textiles for wearable healthcare applications. Proceedings of the 19th World Text Conf. Text Crossroads.

[66] Antonelli A., Guilizzoni D., Angelucci A., Melloni G., Mazza F., Stanzi A., Venturino M., Kuller D., Aliverti A. (2020). Comparison between the Airgo^TM^ device and a metabolic cart during rest and exercise. Sensors.

[67] Romei M., Mauro A.L., D’Angelo M.G., Turconi A.C., Bresolin N., Pedotti A., Aliverti A. (2010). Effects of gender and posture on thoraco-abdominal kinematics during quiet breathing in healthy adults. Respir. Physiol. Neurobiol..

[68] Pacelli M., Caldani L., Paradiso R. (2006). Textile piezoresistive sensors for biomechanical variables monitoring. Annu. Int. Conf. IEEE Eng. Med. Biol. Proc..

[69] Guay P., Gorgutsa S., Larochelle S., Messaddeq Y. (2017). Wearable contactless respiration sensor based on multi-material fibers integrated into textile. Sensors.

[70] Curone D., Secco E.L., Tognetti A., Loriga G., Dudnik G., Risatti M., Whyte R., Bonfiglio A., Magenes G. (2010). Smart garments for emergency operators: The ProeTEX project. IEEE Trans. Inf. Technol. Biomed..

[71] Issatayeva A., Beisenova A., Tosi D., Molardi C. (2020). Fiber-optic based smart textiles for real-time monitoring of breathing rate. Sensors.

[72] Scilingo E.P., Gemignani A., Paradiso R., Taccini N., Ghelarducci B., Rossi D. (2005). De Performance evaluation of sensing fabrics for monitoring physiological and biomechanical variables. IEEE Trans. Inf. Technol. Biomed..

[73] Massaroni C., Tocco J.D., Presti D.L., Longo U.G., Miccinilli S., Sterzi S., Formica D., Saccomandi P., Schena E. (2019). Smart textile based on piezoresistive sensing elements for respiratory monitoring. IEEE Sens. J..

[74] Cala S.J., Kenyon C.M., Ferrigno G., Carnevali P., Aliverti A., Pedotti A., Macklem P.T., Rochester D.F. (1996). Chest wall and lung volume estimation by optical reflectance motion analysis. J. Appl. Physiol..

[75] Husain M.D., Kennon R., Dias T. (2014). Design and fabrication of temperature sensing fabric. J. Ind. Text..

[76] Ten Eyck L.G., Lynam L.E., Xia H., Deng K.-L.J. (2007). Temperature Sensing Fabric. U.S. Patent.

[77] Morris D., Coyle S., Wu Y., Lau K., Wallace G., Diamond D. (2009). Bio-sensing textile with integrated optical detection system for health monitoring. Sens. Actuators B Chem..

[78] Totaro M., Poliero T., Mondini A., Lucarotti C., Cairoli G., Ortiz J., Beccai L. (2017). Soft smart garments for lower limb joint position analysis. Sensors.

[79] Fernández-Caramés T.M., Fraga-Lamas P. (2018). Towards the internet-of-smart-clothing: A review on IoT wearables and garments for creating intelligent connected E-textiles. Electronics.

[80] Rodrigues S., Paiva J.S., Dias D., Aleixo M., Filipe R.M., Cunha J.P.S. (2018). Cognitive impact and psychophysiological effects of stress using a biomonitoring platform. Int. J. Environ. Res. Public Health.

[81] Villar R., Beltrame T., Hughson R.L. (2015). Validation of the Hexoskin wearable vest during lying, sitting, standing, and walking activities. Appl. Physiol. Nutr. Metab..

[82] Villa-Colín J., Shaw T., Toscano W., Cowings P. Evaluation of Astroskin Bio-monitor during high intensity physical activities. Proceedings of the Memorias del Congreso Nacional de Ingeniería Biomédica.

[83] Andreev E., Radeva V., Nikolova M. Innovative biomonitoring systems in the aerospace industry. Proceedings of the Communications, Electromagnetics and Medical Applications Conference 2019.

[84] Sarmento A., Vignati C., Paolillo S., Lombardi C., Scoccia A., Nicoli F., Mapelli M., Leonardi A., Ossola D., Rigoni R. (2018). Qualitative and quantitative evaluation of a new wearable device for ECG and respiratory Holter monitoring. Int. J. Cardiol..

[85] Chronolife SAS. https://www.chronolife.net/.

[86] Emglare Heart. https://emglare.com/.

[87] Katzburg S., Dor-Haim H., Weiss A.T., Leibowitz D. (2019). Detection of unexpected ischaemia due to left main disease during tele-rehabilitation using 12-lead electrocardiogram monitoring: A case report. Eur. Hear. J. Case Rep..

[88] Reyzelman A.M., Koelewyn K., Murphy M., Shen X., Yu E., Pillai R., Fu J., Scholten H.J., Ma R. (2018). Continuous temperature-monitoring socks for home use in patients with diabetes: Observational study. J. Med. Internet Res..

[89] AiQ Smart Clothing. http://www.aiqsmartclothing.com/.

[90] Callejas Sandoval S., Kwon S. Smart wearable technologies to promote safety in aging construction labor. Proceedings of the Creative Construction Conference 2019.

[91] BioSerenity Neuronaute. https://www.bioserenity.com/en/neuro/.

[92] Fouassier D., Roy X., Blanchard A., Hulot J.S. (2020). Assessment of signal quality measured with a smart 12-lead ECG acquisition T-shirt. Ann. Noninvasive Electrocardiol..

[93] Frerichs I., Vogt B., Wacker J., Paradiso R., Braun F., Rapin M., Caldani L., Chételat O., Weiler N. (2020). Multimodal remote chest monitoring system with wearable sensors: A validation study in healthy subjects. Physiol. Meas..

[94] Lynn S.K., Watkins C.M., Wong M.A., Balfany K., Feeney D.F. (2018). Validity and reliability of surface electromyography measurements from a wearable athlete performance system. J. Sports Sci. Med..

[95] Nazari G., Bobos P., MacDermid J.C., Sinden K.E., Richardson J., Tang A. (2018). Psychometric properties of the Zephyr bioharness device: A systematic review. BMC Sports Sci. Med. Rehabil..

[96] Fox J.L., O’Grady C.J., Scanlan A.T., Sargent C., Stanton R. (2019). Validity of the Polar Team Pro Sensor for measuring speed and distance indoors. J. Sci. Med. Sport.

[97] Komodo Technologies Inc. https://komodotec.com/.

[98] Pesola A.J., Laukkanen A., Tikkanen O., Sipilä S., Kainulainen H., Finni T. (2015). Muscle inactivity is adversely associated with biomarkers in physically active adults. Med. Sci. Sport. Exerc..

[99] D’Addio G., Iuppariello L., Bifulco P., Lanzillo B., Pappone N., Cesarelli M. (2017). Validity and reliability of textile system Sensoria for posturographic measurements. G. Ital. Med. Lav. Ergon..

[100] Wearable X. https://www.wearablex.com/.

[101] SUPA. https://www.supa.ai/.

[102] Gerhardt U., Breitschwerdt R., Thomas O. (2018). mHealth Engineering: A Technology Review. J. Inf. Technol. Theory Appl..

[103] Majumder S., Mondal T., Deen M.J. (2017). Wearable sensors for remote health monitoring. Sensors.

[104] Hexoskin. https://www.hexoskin.com/.

[105] Yeung J., Catolico D., Fullmer N., Daniel R., Lovell R., Tang R., Pearson E.M., Rosenberg S.S. (2019). Evaluating the sensoria smart socks gait monitoring system for rehabilitation outcomes. PM R.

[106] L.I.F.E. Multipurpose Wearable Computers. https://www.x10y.com/.

[107] Curone D., Tognetti A., Secco E.L., Anania G., Carbonaro N., De Rossi D., Magenes G. (2010). Heart rate and accelerometer data fusion for activity assessment of rescuers during emergency interventions. IEEE Trans. Inf. Technol. Biomed. Publ. IEEE Eng. Med. Biol. Soc..

[108] Angelucci A., Kuller D., Aliverti A. (2020). A home telemedicine system for continuous respiratory monitoring. IEEE J. Biomed. Heal. Inform..

[109] Huang S., Liu Y., Zhao Y., Ren Z., Guo C.F. (2019). Flexible electronics: Stretchable electrodes and their future. Adv. Funct. Mater..

